# Alcohol abstinence and drinking among African women: data from the World Health Surveys

**DOI:** 10.1186/1471-2458-11-160

**Published:** 2011-03-10

**Authors:** Priscilla Martinez, Jo Røislien, Nirmala Naidoo, Thomas Clausen

**Affiliations:** 1 Norwegian Center for Addiction Research, University of Oslo, Kirkeveien 166, 0450 Oslo, Norway; 2 Department of Biostatistics, Institute of Basic Medical Sciences, University of Oslo, P.O. Box 1122 Blindern, N-0317 Oslo, Norway; 3 Department of Health Statistics and Informatics, World Health Organization, Avenue Appia 20, 1211 Geneva 27, Switzerland

## Abstract

**Background:**

Alcohol use is increasing among women in Africa, and comparable information about women's current alcohol use is needed to inform national and international health policies relevant to the entire population. This study aimed to provide a comparative description of alcohol use among women across 20 African countries.

**Methods:**

Data were collected as part of the WHO World Health Survey using standardized questionnaires. In total, 40,739 adult women were included in the present study. Alcohol measures included lifetime abstinence, current use (≥1 drink in previous week), heavy drinking (15+ drinks in the previous week) and risky single-occasion drinking (5+ drinks on at least one day in the previous week). Country-specific descriptives of alcohol use were calculated, and K-means clustering was performed to identify countries with similar characteristics. Multiple logistic regression models were fitted for each country to identify factors associated with drinking status.

**Results:**

A total of 33,841 (81%) African women reported lifetime abstinence. Current use ranged from 1% in Malawi to 30% in Burkina Faso. Among current drinkers, heavy drinking varied between 4% in Ghana to 41% in Chad, and risky single-occasion drinking ranged from <1% in Mauritius to 58% in Chad. Increasing age was associated with increased odds of being a current drinker in about half of the countries.

**Conclusions:**

A variety of drinking patterns are present among African women with lifetime abstention the most common. Countries with hazardous consumption patterns require serious attention to mitigate alcohol-related harm. Some similarities in factors related to alcohol use can be identified between different African countries, although these are limited and highlight the contextual diversity of female drinking in Africa.

## Background

Alcohol use is an important factor in any woman's health risk profile. Harmful patterns of alcohol consumption are strongly associated with increased morbidity and mortality [1]. Alcohol related morbidities include mental health disorders such as substance dependence and depression, and physical morbidities such as breast cancer, and HIV infection [2-5]. Women also experience unique negative social consequences of alcohol use that impact health, from increased risk of domestic violence and stigma [6,7]. The negative health and social consequences of alcohol use are further moderated by the volume of alcohol consumed and the pattern of use over time [8].

Alcohol use among women in Africa has traditionally been quite low, and high rates of lifetime abstention persist in many African countries [9]. However, population-based surveys have documented rates of alcohol use and harmful drinking among African women that raise concern, including episodic binge drinking and regular high consumption. Prevalence of alcohol use in the past-year among women was estimated at 30% in Bostwana and 47% in Namibia [9,10]. Heavy drinking was found in 38% of women currently drinking in Nigeria and 20% among current female drinkers in Uganda [11,12]. The negative consequences of harmful alcohol use are illustrated by studies that identify women's alcohol use as a risk factor for HIV infection in Uganda and South Africa [13,14]. From the limited evidence available, factors associated with alcohol use among women in low to middle income countries included being single, higher socio-economic status and higher levels of education [15-17].

African countries are categorized as low to middle income, and as such are often limited alcohol-policy environments [18]. As observed in Thailand, such an environment coupled with increasing incomes resulted in pronounced increases in rates of drinking among young women [19]. The heavy influence of the alcohol industry on the development of national alcohol policies favorable to alcohol advertising and distribution has recently been documented in several African countries [20]. The combination of minimally regulated alcohol companies and increased commoditization of their products, along with higher levels of social tolerance towards female drinking predicates increases in the number of African women imbibing alcohol.

A recent study using data from the WHO's World Health Survey observed diverse patterns of drinking among 20 African countries, supporting the contention there is a variety of national drinking habits across the African continent [21]. This study, however, did not explicitly examine patterns of use among currently drinking women. Indeed, there is a paucity of research investigating African women's use of alcohol and associated factors at a country level, limiting our current knowledge of the different ways women consume alcohol in different African countries. This knowledge is important for gauging the expected increase in alcohol use by African women, and the inclusion of women's interests in the development of national health and alcohol policies.

The WHO's World Health Survey provided data on alcohol use and sociodemographics among women in 20 African countries [22]. Using this data, the present study provides a comparative description of alcohol use among women in Africa. We also aimed to identify broad similarities and differences in women's drinking behaviors across the 20 countries, and determine sociodemographic factors associated with current drinking levels and different drinking patterns by country.

## Methods

### Data collection

The data used for this study is publicly available from the WHO. Data were collected as part of the WHO World Health Survey (WHS) between 2002 and 2004 in 20 African countries [22]. Household samples were drawn from nationally representative sampling frames. A stratified, multi-stage cluster design was used where each household had a known non-zero probability of selection. One single respondent aged 18 years or above was randomly selected from each eligible household using Kish tables.

In total 77,165 adults aged 18 years and older were included, and of these, 40,739 (53%) were women. Response rates were reported at both the household and individual level and varied between 54 and 98% at the household level (median = 90%), and 85 and 99% at the individual level (median = 98%) [23].

The WHS used identical questionnaires for the face-to-face interviews in all 20 countries. Individual level data included sociodemographic variables such as marital status, education and employment. WHS protocols and procedures were approved by the ethics committees in each participating country and informed consent was obtained from all participants. The instruments and sampling designs are described in further detail elsewhere [23,24].

### Alcohol data

The question "have you ever consumed a drink that contains alcohol?" was used to identify lifetime abstainers. If the respondent indicated positively, they were asked "how many standard drinks were consumed each day in the past 7 days". From this, we constructed three variables related to drinking: 'current drinkers' were defined as any respondent who consumed at least 1 standard drink in the previous 7 days; 'heavy drinkers' were defined as those who had consumed a total of 15 or more standard drinks during the last 7 days; and 'risky single-occasion drinkers' were defined as those who consumed at least 5 or more standard drinks of alcohol on at least one day of the previous week. Note these three variables are not mutually exclusive. A showcard with pictures was used to illustrate what was meant by a "standard drink", and defined by WHS as containing between 8-13 g of ethanol depending on the country.

### Statistical analyses

All data were weighted, with post-stratification adjustments for age and gender using the UN population estimates as the reference population. Data were stratified by gender, and descriptive statistics presented as frequencies (%) or means (SD). Prevalences for the 'heavy drinker' and 'risky single-occasion drinker' variables are presented out of the total 'current drinker' group, unless otherwise specified. All the rates presented are weighted proportions.

In order to explore whether the 20 countries could be grouped into clusters based on similarities in percentages of the three drinking variables, i.e. 'lifetime abstainers', 'heavy drinkers' and 'risky single-occasion drinkers', we performed K-means clustering, averaging over 25 runs [25]. K-means clustering aims to divide a number of observations into a number of clusters where each observation belongs to the cluster with the nearest mean value(s) and the within-cluster variability is at a minimum. Only the 14 countries with values above 1% for 'heavy drinkers' and 'risky single-occasion drinkers' were included in the K-means clustering analysis. Given this rather small sample size the robustness of the result of the clustering analysis was assessed by removing outliers and removing random data points, i.e. countries, and re-running the analysis in each case [26].

In order to assess possible predictors of the three constructed dependent variables 'current drinking', 'heavy drinking' and 'risky single-occasion drinking', we fitted separate multiple logistic regression models for each of the 14 countries with more than 30 current drinkers. Included explanatory variables in the regression analyses were age, any education or not, currently married or cohabitating, working for pay and rural setting. In order to assess the validity of the linearity assumption in Generalized Linear Models (GLM), e.g. logistic regression, we first fitted Generalized Additive Models (GAM). GAM is a natural extension of GLM allowing for all types of functional relationships between the dependent and the independent variables [27]. By visual inspection of the results from the GAM analyses, age was not linear with respect to the dependent variables in 7 of the 14 countries, but rather piecewise linear. That is, two linear segments separated by a *breakpoint*, where below and above this breakpoint age has different effects on the dependent variable. For the 7 countries where age showed a linear relation to the dependent variable we thus fitted standard multiple logistic regression analyses, whereas for the 7 countries where age showed a piecewise linear relationship to the dependent variable we fitted piecewise linear logistic regression models including estimates of the accompanying breakpoint [28].

Data analysis was performed in STATA 9.0 and R 2.9.0 [29,30].

## Results

In total 33,841 (81%) of the African women from the 20 countries were lifetime abstainers, with rates ranging from 56% in Mauritius to 99% in Comoros (Table 1). A total of 3,592 (10%) women were current drinkers, with the highest national rate in Burkina Faso (30%) and the lowest in Tunisia (<0.1%). Of the entire sample, being a heavy drinker and a risky single-occasion drinker was observed among 584 (1%) and 713 (2%) of the women, respectively, with the highest proportions of heavy drinkers and risky single-occasion drinkers among current drinkers observed in Burkina Faso and Chad. In total, the proportion of risky single-occasion drinkers among heavy drinkers was 70%, ranging from 16% in Ethiopia to 92% in South Africa (data not shown).

**Table 1:** Patterns of drinking among adult women in 20 African countries

Country	n	Lifetime abstainers (%)*	Current drinkers (%)*	Heavy drinkers (%)**	Risky single-occasion drinkers (%)**
**Burkina Faso**	2543	64.4	29.5	33.5	31.0
**Chad**	2435	79.0	17.0	41.3	57.5
**Comoros**	969	99.9	-	-	-
**Congo**	1185	59.4	18.9	5.2	15.3
**Cote d'Ivoire**	1339	73.0	12.1	7.1	6.9
**Ethiopia**	2535	64.1	19.1	5.3	1.8
**Ghana**	2159	63.0	12.9	4.4	3.3
**Kenya**	2537	89.5	4.1	14.0	12.4
**Malawi**	3082	92.8	1.0	11.5	36.4
**Mali**	1749	95.8	2.5	8.6	22.4
**Mauritania**	2193	97.7	-	-	-
**Mauritius**	2016	56.1	11.8	0.9	0.3
**Morocco**	2926	99.8	0.0	-	-
**Namibia**	2379	69.8	22.5	12.1	17.8
**Senegal**	1223	97.7	1.0	13.0	21.4
**South Africa**	1228	82.0	13.5	15.6	30.5
**Swaziland**	1189	92.6	5.0	8.8	18.5
**Tunisia**	2411	99.8	-	-	-
**Zambia**	2088	85.8	5.9	17.7	27.6
**Zimbabwe**	2553	90.8	3.4	7.2	18.3

*% of total

**% of current drinkers

- weighted proportion estimates excluded for countries with 5 or less current drinkers

In Comoros, Mali, Mauritania, Morocco, Senegal and Tunisia more than 95% of women were lifetime abstainers with fewer than 30 current drinkers in this sample. These six countries were thus excluded from further descriptive comparisons, cluster analysis and regression analysis of 'current drinkers', 'heavy drinkers' and 'risky single-occasion drinkers'.

Of the remaining 14 countries, current drinkers ranged from 1% in Malawi to 30% in Burkina Faso, where 12 countries had rates below 20%. Rates of heavy drinkers among current drinkers varied widely, from 4% in Ghana to 41% in Chad, and were below 20% in 12 countries. Rates of risky single-occasion drinkers among current drinkers were below 20% in 9 countries, and ranged from <1% in Mauritius to 19% in Swaziland, whereas those with rates above 20% ranged from 28% in Zambia to 58% in Chad.

**Figure 1:**
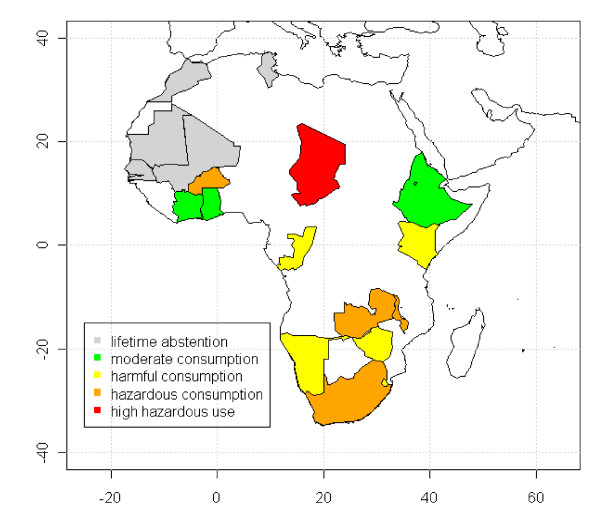
**Geographical distribution of clusters from K-means clustering analysis labeled by drinking pattern and countries with high lifetime alcohol abstention**.

K-means clustering of the three drinking variables produced four clusters of countries (Figure 1); the first cluster included countries with low- to mid-range percentages of lifetime abstainers (56%-73%) and few heavy drinkers and risky single-occasion drinkers (4%-7% and 2%-7%, respectively) and was labeled "moderate consumption countries"; the second cluster included countries with a mid- to high-level range of lifetime abstinence (60%-93%) and somewhat more heavy drinkers and risky single-occasion drinkers (5%-12% and 12%-19%, respectively) and was labeled "harmful consumption countries"; the third cluster was also made up of countries with a mid- to high-level range of lifetime abstention (64% to 93%), but with higher heavy drinker and risky single-occasion drinker rates (12%-34% and 28%-36%, respectively) and thus labeled "hazardous consumption countries"; the fourth and final cluster represents Chad only, which has a moderate lifetime abstention rate of 79% and is a high outlier on the proportion of heavy drinkers and risky single-occasion drinkers (41% and 58%, respectively). Of the 14 countries included, 4 were in the "moderate consumption" cluster, 5 in the "harmful consumption" cluster and 4 in the "hazardous consumption" cluster.

Sociodemographic variables by current drinker status for each of the 20 countries are presented in Table 2. Current drinkers in all countries were either roughly the same age or older than lifetime abstainers, except for Congo. For all countries except Swaziland, an equal or higher proportion of current drinkers were working for pay than lifetime abstainers.

**Table 2:** Selected sociodemographics by drinking status among women in 14 African contries

Country n		Age Mean (SD)	Any education (%)	Working for pay (%)	Married/ cohabitating (%)	Rural setting (%)
**Burkina Faso**						
current drinkers	669	39.6 (15.1)	7.4	34.3	82.2	91.7
lifetime abstainers	1699	34.1 (13.6)	9.8	34.6	88.7	82.3
**Chad**						
current drinkers	358	35.6 (13.1)	23.6	59.3	71.8	82.7
lifetime abstainers	1986	34.6 (14.8)	14.4	49.3	77.1	80.2
**Congo**						
current drinkers	248	33.4 (12.3)	92.9	40.7	59.3	6.8
lifetime abstainers	721	35.3 (14.8)	84.0	34.0	46.9	6.3
**Cote d'Ivoire**						
current drinkers	146	38.2 (14.3)	52.0	51.8	53.0	35.1
lifetime abstainers	1025	34.2 (14.0)	53.9	48.6	56.2	30.3
**Ethiopia**						
current drinkers	465	37.2 (15.6)	19.4	39.9	68.7	90.6
lifetime abstainers	1655	34.8 (14.8)	38.5	32.6	65.3	87.3
**Ghana**						
current drinkers	303	42.7 (15.8)	58.8	86.5	66.1	66.0
lifetime abstainers	1334	39.2 (16.6)	61.2	77.0	59.1	53.4
**Kenya**						
current drinkers	134	40.7 (17.1)	66.5	64.1	58.0	67.3
lifetime abstainers	2216	34.6 (14.1)	87.1	50.4	64.9	83.0
**Malawi**						
current drinkers	45	52.0 (14.1)	66.3	44.7	41.8	94.1
lifetime abstainers	2851	34.0 (15.5)	68.4	33.1	69.2	91.4
**Mauritius**						
current drinkers	244	42.9 (14.0)	90.4	42.9	74.2	51.2
lifetime abstainers	1112	41.6 (15.9)	88.3	31.4	66.0	55.5
**Namibia**						
current drinkers	494	38.3 (16.4)	73.2	25.0	31.0	70.9
lifetime abstainers	1681	38.2 (18.1)	79.6	25.7	33.3	62.0
**South Africa**						
current drinkers	168	42.3 (17.8)	86.3	33.3	36.4	36.8
lifetime abstainers	999	37.7 (15.5)	91.6	31.7	35.9	46.1
**Swaziland**						
current drinkers	59	50.6 (14.9)	33.3	10.6	70.4	85.9
lifetime abstainers	1108	37.5 (16.1)	78.1	22.5	51.5	73.5
**Zambia**						
current drinkers	115	42.2 (17.6)	73.3	54.9	56.2	53.5
lifetime abstainers	1799	34.0 (15.5)	79.3	45.7	58.2	65.5
**Zimbabwe**						
current drinkers	85	44.9 (15.3)	82.0	20.0	68.3	61.2
lifetime abstainers	2346	35.9 (15.6)	88.8	19.5	59.8	60.7

In multiple logistic regression analyses to identify factors associated with being a current drinker, increasing age was associated with increased odds in 9 of the 14 countries (Table 3). In countries with a piecewise linear relationship between age and current drinker status, the breakpoint for the change of the effect of age ranged from 33 to 54 (median = 49). In 4 of these countries, an increase in age was significantly associated with increased odds of being a current drinker before the breakpoint only; for ages above the breakpoint increasing age was not associated with increased odds of being a current drinker. Of the 5 countries for which having any education was a statistically significant predictor, it decreased the odds of being a current drinker except in Chad. Working for pay was significantly associated with an increased odds of being a current drinker in Mauritius, Chad and Ghana, while being married/cohabitating was associated with a decreased odds of being a current drinker in 4 of the 5 countries for which it was statistically significant. Living in a rural setting was also associated with a decreased odds of being a current drinker in 2 of the 3 countries for which it was statistically significant.

**Table 3:** Multiple logistic and piecewise regression results for current drinking among women in 14 African countries

Countries with linear effect of age	Age OR (95% CI)	Any education OR (95% CI)	Working for pay OR (95% CI)	Married/co-habiting OR (95% CI)	Rural setting OR (95% CI)
**Congo**	1.00 (0.98-1.01)	2.01 (0.83-4.87)	1.30 (0.59-2.89)	1.41 (0.89-2.22)	1.05 (0.76-1.46)
**Ethiopia**	1.00 (0.99-1.01)	0.39 (0.28-0.57)***	1.28 (0.95-1.71)	0.76 (0.61-0.96)*	1.00 (0.78-1.36)
**Malawi**	1.05 (1.04-1.07)*	2.13 (0.85-5.39)	1.45 (0.72-2.94)	0.55 (0.27-1.10)	1.11 (0.74-1.68)
**Mauritius**	1.02 (1.00-1.03)**	1.31 (0.68-2.53)	1.74 (1.26-2.42)***	1.55 (1.06-2.25)*	0.92 (0.68-1.25)
**South Africa**	1.01 (0.99-1.02)	0.66 (0.38-1.14)	1.32 (0.93-1.88)	1.21 (0.86-1.70)	0.84 (0.71-0.99)*
**Zambia**	1.04 (1.02-1.05)***	1.00 (0.53-1.88)	1.50 (0.90-2.50)	1.05 (0.63-1.75)	0.71 (0.54-0.92)*
**Zimbabwe**	1.04 (1.02-1.06)***	1.07 (0.54-2.12)	1.19 (0.62-2.29)	1.60 (0.92-2.78)	0.87 (0.63-1.21)

Countries with piecewise linear effect of age	**OR (95% CI) before breakpoint** **OR (95% CI) after breakpoint** ***Age at breakpoint (95% CI)***				

**Burkina Faso**	1.03 (1.02-1.05) *** 0.99 (0.96-1.03) *53 (38-69)*	1.22 (0.88-1.69)	1.13 (0.90-1.41)	0.70 (0.53-0.94)*	2.39 (1.85-3.08)***
**Chad**	1.03 (0.99-1.06) 0.95 (0.93-0.97) *37(29-46)*	2.33 (1.62-3.35)***	1.68 (1.24-2.27) ***	0.53 (0.38-0.75) ***	1.42 (0.96-2.12)
**Cote d'Ivoire**	1.04 (1.01-1.08) * 0.99 (0.94-1.03) *49 (32-65)*	1.26 (0.78-2.02)	0.92 (0.59-1.43)	0.76 (0.48-1.20)	1.33 (0.84-2.09)
**Ghana**	1.02 (1.00-1.04) * 0.99 (0.97-1.02) *51 (28-74)*	0.85 (0.62-1.15)	2.00 (1.29-3.12)**	0.92 (0.69-1.24)	1.29 (0.97-1.73)
**Kenya**	1.08 (0.99-1.19) 0.99 (0.97-1.01) *33 (21-45)*	0.43 (0.26-0.69)***	0.98 (0.65-1.47)	0.76 (0.51-1.15)	0.76 (0.50-1.17)
**Namibia**	1.03 (1.00-1.05) 0.97 (0.95-0.98) ** 41 (34-48)	0.59 (0.43-0.79)***	1.05 (0.82-1.34)	0.66 (0.52-0.84)***	1.18 (0.94-1.48)
**Swaziland**	1.06 (1.02-1.10)** 0.98 (0.92-1.05) 54 (36-72)	0.43 (0.20-.92)*	0.90 (0.40-2.05)	0.76 (0.39-1.51)	1.53 (0.61-3.83)

* p ≤ 0.05; ** p ≤ 0.01 ***p ≤ 0.001

Regression analysis with heavy drinker and risky single-occasion drinker as the dependent variable revealed very few statistically significant covariates (data not shown).

There were no consistent patterns in the significance, magnitude or direction of the covariates within the country clusters generated by the K-means clustering.

## Discussion

Our findings show widespread lifetime abstention from alcohol use among women in 20 African countries, limited though existing heavy and risky single-occasion drinking, and no firm geographic distribution of drinking patterns across the countries examined. Among currently drinking women our findings show moderate alcohol consumption is the most common pattern, and being a risky single-occasion drinker is more common than being a heavy drinker. The results further indicate drinking increases with age in several countries, where in some countries it clearly stabilizes or declines after mid-life.

The predominance of lifetime abstention from alcohol support female drinking is not a common or accepted part of African culture, likely due to religion, cultural tradition and gender roles [31]. This "alcohol naivete" presents an opportunity to establish and promote healthy drinking habits among the vast majority of African women. In Europe, the pattern of drinking in a population has been shown to be stable over time even when total consumption varies [32]. If also true in Africa, establishing healthy patterns of alcohol consumption among the majority of women would serve the public health of African countries far into the future. This effort would be particularly relevant and timely given the current expansion of the alcohol industry in Africa. Women are a large portion of the population available for recruitment into regular drinking and will also benefit from improved economic situations, creating a "perfect storm" for an increase in alcohol use and related harm [33]. Such circumstances and increases in hazardous drinking among women have already been observed in Brazil and India [34]. While these observations are not directly applicable to Africa due to the difference in context and there are no direct reports of increased harmful use among women due specifically to improved economic status and increased alcohol availability in Africa, documentation of increased use and a report of weak alcohol policies suggest this scenario possible in many African countries [11,13,15,20]. These observations and the potential of such in Africa lend support for calls encouraging national action through global coordination and an international health policy [35].

Risky single-occasion drinking as the more common pattern of harmful use highlights the need for evidence-based, cost-effective interventions to avoid the development of alcohol related harm in countries where hazardous drinking patterns are observed. Indeed, there have already been calls from the WHO for Africa to address the harmful use of alcohol, and concerns from South Africa and Uganda about hazardous drinking habits among their female citizens [36-38].

The clusters of countries classified with "moderate consumption", "harmful consumption" and "hazardous consumption", with Chad in a position on its own, provide further evidence different types of drinking behaviors are present among female drinkers in Africa and a diversity of drinking patterns is common across the African continent. While there may be similar rates of a single drinking pattern between clusters, rates of the other drinking measures may be quite different, and these differences would have implications for differences in national and regional alcohol policies and interventions. The countries with the highest lifetime abstention rates, which were excluded from the cluster analysis, are located in the northern part of Africa and are predominantly Muslim. These results are consistent with other studies and the Islamic doctrine [9,21]. However, this does not apply to all the countries with high lifetime abstention rates, e.g. Zimbabwe or Malawi; these countries are neither predominantly Muslim nor located in northern Africa. No other consistent geographic distribution of countries by cluster are evident, highlighting the importance of local culture and tradition on female drinking behavior, and limited transfer of drinking habits across country borders.

Sociodemographic factors associated with current drinking varied across the 14 countries analysed, further reflecting the contextual diversity of alcohol use among African women. Older age was significantly associated with current drinking in 8 countries, potentially reflecting higher levels of independence and authority conferred by older age. The significant, non-linear relationship between age and current drinking observed in 5 countries is consistent with the idea of changes in drinking behavior over the lifespan, where drinking increases as age increases and then stabilizes or decreases around mid-life. This dynamic association indicates the opportunity for prevention interventions among younger age groups.

Having any education reduced the likelihood of current drinking in four of the five countries for which it was statistically significant save Chad. This finding is inconsistent with studies from Europe that observe an association between higher levels of education and drinking among women [17,39]. However, this association is reasonable given the differences in context and availability of education between Europe and Africa as a whole. Women receiving *any *education in Africa likely represent a different demographic than those receiving any or higher education in a Western context.

Working for pay was significantly associated with an increase in current drinking in Mauritius, Chad and Ghana. Not being married or cohabitating increased the likelihood of current drinking for four of the five countries for which it was statistically significant. Having one's own disposable income and being single have been identified as factors in women's alcohol consumption in resource rich and resource poor settings, and these factors may become more common and pertinent with economic development [40].

The lack of consistent, significant associations between sociodemographic factors across all the countries and within the country clusters emphasizes the importance of local, tailored alcohol assessments in each country to ensure relevance and utility.

### Methodological considerations

The estimates of being a current drinker, risky single-occasion drinker and heavy drinker are likely underestimates of the true value. Stigma, religious beliefs, social norms and gender roles may contribute to the underreporting of alcohol use [41]. Conversely, these same reasons may lead to the overreporting of lifetime abstention. There may also be reduced recall of lifetime consumption when alcohol use among women is socially sanctioned at infrequent but common festivities, e.g. birthdays. We have no means of adjusting for potential under- or over-reporting of alcohol consumption, or whether this source of potential misclassification is equally distributed between the included countries. Also, the volume of alcohol consumed was self-reported and may add to variation in the number of "standard drinks" consumed. Both volume and concentration of alcohol consumed may be especially difficult to estimate where homebrews are common. The reported prevalences of all drinking patterns for all countries should thus be read with caution.

The current drinker, heavy drinker and risky single-occasion drinker measures were based on a 1-week recall which may increase the precision of the amount of drinks consumed but compromise the accuracy of the measurement of women who are true "current drinkers", "heavy drinkers " or " risky single-occasion drinkers", as a 7-day recall may not necessarily reflect typical drinking behavior. Moreover, this sample contained no data on women who reported ever having a drink but no consumption in the last week, excluding a group of potential "current" drinkers, or heavy or risky-single occasion drinkers who did not drink in the previous week. Finally, no personal income variable was available in this dataset, and as noted, income is an important factor to consider in women's drinking behavior and should be purposefully included in future studies. Indeed, any variables measuring context-specific aspects of African women's use of alcohol would be useful.

This paper nonetheless has important value as it covers more than a third of African countries and contributes important knowledge for describing the drinking behavior of women in Africa, and can serve as a baseline against which to measure drinking patterns in the future.

## Conclusions

A variety of drinking patterns are present among African women with lifetime abstention being the most common. The identification of countries with hazardous consumption patterns demands immediate and serious attention to avoid and mitigate alcohol-related harm. Some similarities in factors related to alcohol use can be identified between different African countries, although these are limited and diversity prevails. Further investigations are required to understand the country-specific context of female drinking in Africa and to tailor national alcohol policies.

## Competing interests

The authors declare that they have no competing interests.

## Authors' contributions

PM, TC and NN contributed to the design of the study. NN contributed to data collection and data management. PM led the writing and carried out statistical analysis. JR contributed to the statistical design and analysis. All authors read and approved the final manuscript.

## Pre-publication history

The pre-publication history for this paper can be accessed here:

http://www.biomedcentral.com/1471-2458/11/160/prepub
